# Flatfeet Severity-Level Detection Based on Alignment Measuring

**DOI:** 10.3390/s23198219

**Published:** 2023-10-02

**Authors:** Fatmah A. Alsaidi, Kawthar M. Moria

**Affiliations:** 1 Department of Computer Science, King Abdulaziz University, Jeddah 21589, Saudi Arabia; 2 Department of Electrical and Computer Engineering, King Abdulaziz University, Jeddah 21589, Saudi Arabia

**Keywords:** flat foot, template matching, Convolutional Neural Network, Random Forest, VGG-16, lateral Calcaneal Inclination Angle, lateral Meary’s angle, lateral Arch Angle

## Abstract

Flat foot is a postural deformity in which the plantar part of the foot is either completely or partially contacted with the ground. In recent clinical practices, X-ray radiographs have been introduced to detect flat feet because they are more affordable to many clinics than using specialized devices. This research aims to develop an automated model that detects flat foot cases and their severity levels from lateral foot X-ray images by measuring three different foot angles: the Arch Angle, Meary’s Angle, and the Calcaneal Inclination Angle. Since these angles are formed by connecting a set of points on the image, Template Matching is used to allocate a set of potential points for each angle, and then a classifier is used to select the points with the highest predicted likelihood to be the correct point. Inspired by literature, this research constructed and compared two models: a Convolutional Neural Network-based model and a Random Forest-based model. These models were trained on 8000 images and tested on 240 unseen cases. As a result, the highest overall accuracy rate was 93.13% achieved by the Random Forest model, with mean values for all foot types (normal foot, mild flat foot, and moderate flat foot) being: 93.38 precision, 92.56 recall, 96.46 specificity, 95.42 accuracy, and 92.90 F-Score. The main conclusions that were deduced from this research are: (1) Using transfer learning (VGG-16) as a feature-extractor-only, in addition to image augmentation, has greatly increased the overall accuracy rate. (2) Relying on three different foot angles shows more accurate estimations than measuring a single foot angle.

## 1. Introduction

### 1.1. Background

Flat foot (Pes Planus) is a common foot deformity in which the arch of the foot is depressed and the plantar part of the foot is either completely or partially contacted with the ground [1]. Mostly, flat feet can affect the natural gait and usually require therapy to resolve; in severe cases where the pain grows to be unbearable, surgical intervention is required [2,3,4]. According to the same sources, the severity levels of flat feet can be classified into three levels: mild, moderate, and severe (rigid). 

Recently, measuring foot angles from X-ray images has become a preferable scheme for flat foot assessments as it is more affordable to many clinics than using specialized devices [5,6]. During imaging the foot, it is crucial for the foot to be in a weight-bearing state during the imaging, that is, when the foot is bearing the body weight, so the foot angles can be correctly measured [7].

**Figure 1:**
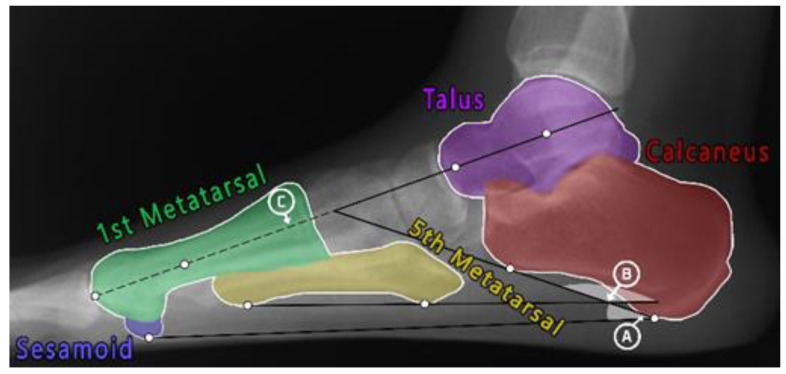
A total of nine PoIs (white dots) and three foot angles: (A) Calcaneal Inclination Angle, (B) Arche Angle, and (C) Meary’s Angle. The figure also shows five bones of interest: Talus (purple), Calcaneus (red), first Metatarsal (green), fifth Metatarsal (yellow), and Sesamoid (blue).

When assessing flat feet from X-ray images, several Points of Interest (PoIs) should be hypothetically allocated and connected to form different foot angles. These angles are then measured and compared at certain intervals to precisely estimate the flatness of the foot as well as its severity level. According to the literature, there are many different foot angles that can be used in diagnosing flat feet; some of these angles can be measured from a certain view, such as a lateral X-ray view where the smallest toe is clearly visible [8,9,10]. Three of the most commonly used foot angles in adult flat foot assessment are: Calcaneal Inclination Angle (i.e., Calcaneal Pitch), Arch Angle (i.e., foot arch), and Meary’s Angle (i.e., Talus-First Metatarsal Angle), as shown in Figure 1 [11,12,13,14,15]. The figure shows nine PoIs allocated (white dots). The angles formed by connecting these points are: (A) the Calcaneal Inclination Angle (CIA), which measures the tilting degree of the Calcaneus bone, aka the heel (in red), using the Sesamoid bone as its base (in blue); (B) the Arch Angle (AA), which measures the tilting degree of the fifth Metatarsal, aka the smallest toe (in yellow); and (C) Meary’s Angle (MA), which measures the tilting of both the Talus (in purple) and the first Metatarsal bones (in green).

These resulting angles are then measured and compared to angle intervals to estimate the flatness of the foot as well as how severe the flatness is (either mild or moderate). Table 1 shows the angle intervals for each of the three aforementioned angles. For the CIA, several sources [16,17,18,19,20,21] agreed that if the angle a was between 20 and 30 degrees, then it was considered a normal foot. Other sources agreed that [16,18,20] if the angle was in the range of 10 to 20 degrees, then it shows a sign of a mild flat foot. Lastly, if the angle was lower than 10 degrees [16], then it is a moderate case of flat foot. When using the AA, any angle that falls between 150 and 165 is a normal foot, while an angle larger than that but smaller than 180 degrees is a mild flat foot. A moderately flat foot has an angle larger than 180 degrees, as stated by [16]. According to the sources [20,22], if the MA was between 0 and 4 degrees, then the foot is normal; if the angle was larger than 4 and smaller than 15, then it is a flat foot case; and lastly, if the angle was larger than mentioned, then it is a moderately flat foot.

### 1.2. Related Works

According to the literature, allocating PoIs can be grouped into traditional image analysis approaches and machine learning-based approaches. Mainly, using the traditional image analysis technique involves defining bone edges and then implementing different mathematical equations to search along the bone edges for the PoI location. The system proposed by Jian et al. [23] measures foot Arch Angle to detect flat feet from lateral X-ray images. The method started with outlining the foot edges of each image using Canny Edge detection, and the noise was reduced by Gaussian blur filters. The image is then divided into four regions based on the percentage of each foot. Within each region, the bone edges are searched for a PoI using different novel mathematical algorithms. The PoIs are then connected, and the foot Arch Angle is measured for a final diagnosis. The proposed method requires direct human supervision to prevent misallocating the PoIs; therefore, the method provides manual adjustment for re-locating the PoIs. The authors performed statistical analysis rather than measuring performance using common performance metrics (such as accuracy, precision, recall, etc.). Yang et al. [24] used Mutual Information (MI) to register the bones of interest (Calcaneal bone and fifth metatarsal bone) for measuring the Arch Angle. MI is used to measure similarities between a template image and a reference image; the higher MI values indicate that both images are correctly aligned. In the method, the template images were created by selecting a representative image of the bones of interest and rotating it to be aligned horizontally against the surface. To measure the angles, the template bones are repeatedly rotated, and the MI is calculated until maximized. The amount of rotation made indicates the AA value, and then the angle defines a flat foot from a normal foot. The accuracy rate was calculated as the hit ratio, achieving 96% accuracy. Kao et al. [25] proposed a method to detect flat feet by measuring Arch Angle. The authors used Projection Profile, which is used to find the total sums of white pixels along each axis, to segment the images into two RoIs consisting of the bones of interest (the Calcaneal and fifth Metatarsal bones). The lower edges of each region were searched for two pixels of minimum spatial location; these PoIs were then connected, and the Arche Angle was measured to determine flat foot. As a result, the method was able to correctly detect 73.33% of the total cases. The authors suggested that measuring additional foot angles besides the Arch Angle would increase the overall accuracy. These traditional image analysis approaches are still in use in many medical imaging-oriented systems. However, from the reviewed results, such approaches may encounter difficulties when the new image differs from the tested ones; therefore, several restricted image enhancements are required to match the original image. Furthermore, these works did not provide a comprehensive comparison with state-of-the-art techniques, nor did they provide detailed results of their work for future comparison. In the proposed work, the new images would require slight image enhancements (such as brightness and contrast) only when needed. Moreover, the proposed method detects three different foot angles and distinguishes between two flat foot severity levels; it provides detailed results, as shown in Section 3.

Machine learning-based approaches consider generalization of the models to adapt new input images that may slightly differ from the trained dataset; additionally, it saves time and effort as well as providing consistent results. The work proposed by [26] deployed Extremely Randomized Forests to detect flat feet by measuring each of the Calcaneal Inclination Angle, Meary’s Angle, and Talar Declination Angle. The input images were searched in a pixel-by-pixel manner for PoIs using 40 Extremely Randomized Trees. In feature extraction, the model searched the images for candidate pixels as PoIs. The voting system was performed twice: once during down-sampling pixels and again after the original resolution was retrieved. The final PoIs were allocated by finding the mean position of candidate points. In classification, the trees are weighted based on the probability of each pixel being a PoI. These probabilities are estimated by the pixel’s spatial position plus its vicinity, including the three-pixel radius around this pixel. The closer the pixel to the PoI position, the higher its probability. To speed up the searching process, each image was divided into four subimages to be searched in parallel. In the proposed work, instead of searching the image using the pixel-by-pixel method, Template Matching is used to quickly identify the potential locations of PoIs, and then the classifier will decide the exact location of the points.

Nitris et al. [27] exploited a transfer learning scheme to overcome the problem of the limited dataset they have. The authors used the ResNet50 model, which was pre-trained on the ImageNet dataset, as the feature extractor for their dataset, and then they added a novel fully Convolutional Neural Network with Adam optimization. The classifier segments the images into three non-overlapping regions, where each region has one PoI located at the exact center of the box. As a result, the average of the differences between manual angular measurements and angles measured by their work was 1.27 degrees. Inspired by the work by [27], in the proposed work, VGG-16 (which was also pre-trained on the ImageNet dataset) is used as a feature extractor only to overcome the problem of a limited dataset. After that, a different classifier determines the exact location of PoIs using these feature vectors.

The latest attempt was proposed by Lauder et al. [28]. Their work provided an experimental set-up for two models: (1) Random Forest regression-voting Constrained Local Models [29], and (2) Spatial Configuration-Net [30]. The experiment’s aim was to detect flat feet from X-ray images and classify them into mild, moderate, and severe cases. The detection is based on allocating 61 PoIs that are used to measure the Calcaneal Inclination Angle, Meary’s Angle, and Cuboid Height. The first model extracts the feature values of each pixel and feeds them to the regressor to determine the most likely position of this pixel. Each tree in the forest was trained independently and voted based on given information from neighboring pixels. Furthermore, all votes are accumulated to predict the position of each pixel, creating a 2D histogram of votes. The second model defines the PoI location based on other PoI locations; it uses a network architecture that predicts each PoI using a combination of its local appearance plus the special configurations of the rest of the PoIs. The output is heatmap regression for each of the 61 PoIs instead of absolute coordinates. The smallest point-to-point error was 2.2 mm achieved by the first model. This work was the only attempt that combined several foot angles for detecting flat feet; however, the authors did not clarify whether there were disagreements between the forementioned angles or not.

As a summary, the problem of automatically detecting flat feet from X-ray images has attracted the interest of the researchers. Driven by the related works, the detection process mainly consists of two main steps: first, a set of PoIs must be allocated and then connected to form a foot angle. Second, this angle is measured and compared to the angle intervals to determine the flatness of the foot. The two most widely used models for allocating PoIs were the Convolutional Neural Network (CNN) and Random Forest (regression). However, a large-scale dataset is required for training these models from scratch. The traditional image analysis approaches that used mathematical equations rather than machine learning approaches did not require large collections of datasets, although they were time-consuming and required several restricted image enhancements before implementing the methods. The work proposed in [27] used transfer learning as a solution for a limited dataset size and proved to achieve satisfying results.

According to the resource [31], manually allocating the PoIs and measuring the resulting foot angles could greatly consume effort and time. Moreover, it is prone to error since the judgment of each practitioner may differ, leading to a final misdiagnosis. Moreover, according to the literature, there was no research that detected flat feet and found their severity levels using a combination of foot angles instead of relying on only a single angle.

For these reasons, the purpose of this work is to assist doctors and practitioners in automatically detecting flat foot deformity from X-ray images by allocating the set of PoIs and finding their coordinates on the image using a classifier. Using these coordinates, the points are connected, and the angle is formed. This process is repeated to form three different foot angles: CIA, AA, and MA, using a total of nine allocated PoIs. The final diagnosis (and the severity level if it was a flat foot) is determined by a combination of these angles votes; whenever an angle results in a different diagnosis than the others, the diagnosis with the highest vote is selected.

## 2. Materials and Methods

**Figure 2:**
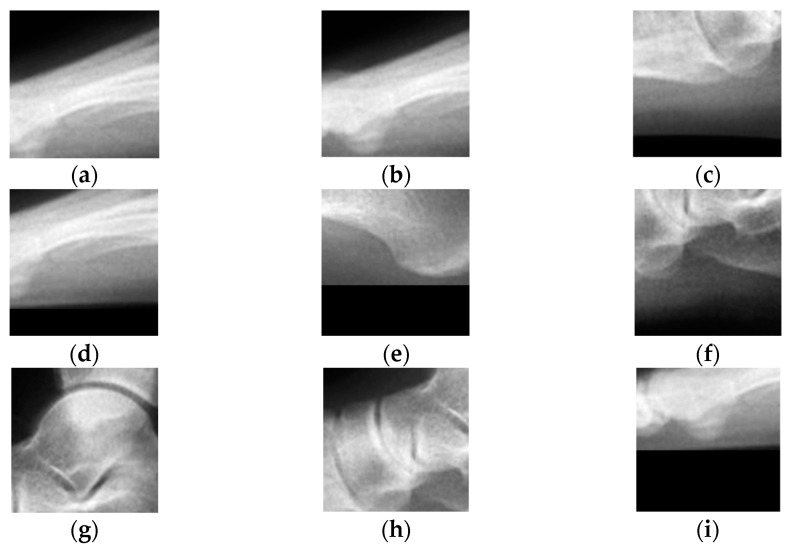
Shows 1 example of a template image for each of the 9 PoIs: (**a**) 1st PoI of 1st metatarsal bone, (**b**) 2nd PoI of 1st metatarsal bone, (**c**) 1st PoI of 5th metatarsal bone, (**d**) 2nd PoI of 5th metatarsal bone, (**e**) 1st PoI of calcaneus, (**f**) 2nd PoI of calcaneus, (**g**) 1st PoI of talus, (**h**) 2nd PoI of talus, and (**i**) the PoI of sesamoid.

Mainly, there are two stages: PoI allocating and angular measurements. To allocate PoIs, a template set is created consisting of several image parts that show potential locations for every PoI. Figure 2 shows 1 example for every 9 PoIs that are used in this research. Using these templates, the input image is searched for any image-part match. These image parts are then fed to the classifier to classify each image part into qualified and disqualified ones based on its likelihood; after that, only a single image part for every PoI is selected as output based on the highest likelihood. Finally, the coordinates of PoIs are extracted from these selected image parts and used to form an angle. Each angle is then measured and compared to the angle intervals to reach an estimation. Since there is a possibility for the angles to disagree in their judgment, the final estimation will be based on a majority vote.

### 2.1. PoI Allocation

The template set consists of image parts that represent each of the nine PoIs individually. These image parts are manually selected and cropped with dimensions of 100 × 100 px, of which the PoI is located at its exact center. Template Matching [32] is used to search the whole input image for any image part with a thresholding match. The searching process involves simply sliding the template image as a window over the input image while calculating the similarity between them. The similarity computation is applied to the matrix representation of the images; it involves multiplying the two metrices and summing the results. Since the output of the matching process is a large set of image parts, a classifier model is used to classify each image part into either qualified or disqualified images for each PoI. To do so, the model is trained to learn the features of each image part to predict a continuous value, interpreting the probability of each image part belonging to a PoI. Furthermore, only a single image part is selected based on the highest predicted likelihood for each PoI. In the case of multiple image parts with the highest predicted likelihood, the first one is selected. With this, for each input image, there is an exact number of image parts equal to the number of PoIs. For comparison, two different classifier models are built and evaluated using the same learned feature vectors, in which the feature extraction part is the same for each model while the classifier part differs. 

The first model is a Convolutional Neural Network (CNN) classifier. According to the literature, the Convolutional Neural Network (CNN) outperformed most machine learning approaches in many medical imaging tasks [14]. The structure of CNN reflects the biological perception of the human brain; it consists of several layers of stacked neurons that are activated with brain signals [13]. Naturally, a CNN structure consists of several pairs of convolutional layers and pooling layers. The convolutional layers use a collection of digital filters to perform convolution operations on the input, while the pooling layers are used to reduce the dimensions (parameters used during backpropagation and the operations performed on a two-dimensional plane) and decide the threshold to avoid overfitting. Several fully connected layers are usually placed between different layer sets to connect every neuron from one layer to every neuron in the next layer. The classification layer is finally used to select the class (or label) that has the highest computed probability. In each layer, an activation function is used to compute the input values of a layer into output values, which are then passed to the next layer as input values again. After compiling the layers, an optimizer is assigned to change the learning rate and weights of neurons to reach the minimum loss function. A higher learning rate makes the model learn faster, but it may miss the minimum loss function. A lower learning rate gives a better chance of finding a minimum loss function. As a tradeoff, a lower learning rate needs higher epochs, which increases time and memory costs. Batch size contributes to making the model learn faster; it refers to assigning batches of training data to the model rather than using all the data at the same time. The smaller batch size means a faster learning process at the cost of increasing the variance of the validation dataset’s accuracy. A larger batch size has a slower learning process, but the validation dataset accuracy has a lower variance. The number of epochs refers to the number of times the training dataset is passed forward and backward through the model. Fewer epochs may increase the risk of underfitting because a model has not learned enough. However, a larger number of epochs may lead to overfitting, for which the model cannot predict new unseen data well enough.

The second model is the Random Forest classifier [33], a supervised machine learning algorithm that uses a large set of trees that operate as an ensemble for classification tasks. The forest consists of a group of individual decision trees created from a randomly selected set of the training set; then, the votes are collected from all the trees to decide the final estimation. Random Forest has two main parameters that affect the model’s performance during training: the number of trees and the depth of the decision trees. The number of decision trees (n_estimators) defines the number of decision trees in the model. Using an increasing number of trees in a forest helps with generalization at the cost of increasing computational time [12,16,17]. The depth of the decision trees defines the maximum height the decision tree can grow inside the forest. The deeper the trees go, the more accurate the model becomes; however, the risk of overfitting also increases.

### 2.2. Angular Measurement

The coordinate of PoI is located at the exact center of the resulted image parts from the classifier: $PoI_{x}=S_{x}+50,\;PoI_{y}=\;S_{y}+50$, where (*S_x_*, *S_y_*) is the starting point of the current image part, and the center of the current image part is the starting point plus 50 pixels. Furthermore, depending on the foot angle to measure, *PoIs* are used to draw the lines that form the foot angles; the angle is then measured using the equation:

$$tan\theta =\left|\frac{m_{2}-m_{1}}{1+\left(m_{2}\times m_{1}\right)}\right|$$

where *m*_1_ and *m*_2_ are the slopes of the first line and the second line, respectively. The slope *m* can be defined using *PoI* pair coordinates:

$$m=\frac{y_{2}-y_{1}}{x_{2}-x_{1}}$$

The angles are then compared to the angle intervals (Table 1) to decide the estimation of the foot. Since the estimations from each angle may differ, the final estimation is the one that most of the angles agree on. The voting method uses a counter to count the number of votes for each estimation and stores them in a dictionary. For example, when two of the angles vote for formal foot and only one foot votes for mild flat foot, then the counter value for normal foot is 3, and the counter values are 1 for mild flat foot and 0 for moderate flat foot. Furthermore, the maximum number of votes is selected, and the corresponding estimation is printed.

## 3. Results

All experiments were implemented in Python programming language version 3.7, TensorFlow 2.2.0, and Keras library 2.3.1. Using a Tesla T4 15.106 GB GPU and an Intel(R) Xeon(R) CPU at 2.20 GHz.

The dataset used consisted of 80 lateral weightbearing X-ray foot images consisting of 30 normal cases, 30 mild flat foot cases, and 20 moderate flat foot cases. The images were collected from King Abdul Aziz University’s hospital and annotated by two radiology specialists. The angles were manually measured (in degrees) using RadiAnt DICOM viewer software (version 2020.1.0). The measured angles are MA, CIA, and AA. A total of nine PoIs were allocated corresponding to the forementioned angles: four PoIs for MA; five PoIs for AA mutually with CIA. Additionally, the severity levels of flat foot cases were annotated for each image as either mild or moderate. The dataset excluded any images that have foot pathologies other than flat foot, as well as images that do not show a full view of bones of interest, such as part of the Calcaneal bone. For testing the models, 77 X-ray images were retrieved from the Lower Extremity Radiographs (LERA) dataset, a public dataset by Varma et al. [34] that consists of normal and abnormal foot, knee, ankle, and hip images collected at Stanford University Medical Center between 2003 and 2014. The dataset is publicly available at https://aimi.stanford.edu/lera-lower-extremity-radiographs URL (accessed on 11 September 2022). The retrieved dataset for this research consists of 29 normal foot, 24 mild flat foot, and 24 moderate flat foot cases.

**Figure 3:**
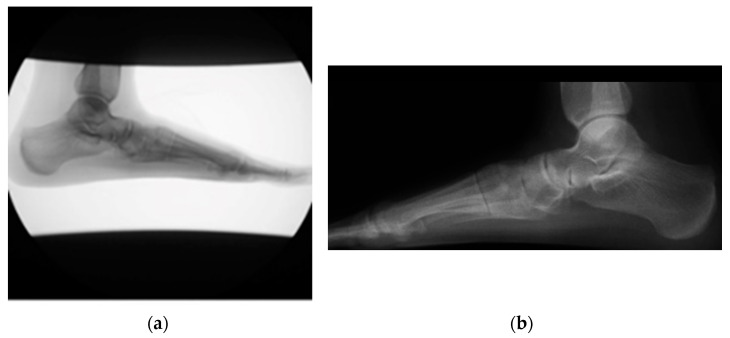
(**a**) An input X-ray image example of the right foot. The radiograph shows a white background, and the foot is not in the center of the image. (**b**) The same input image example from (**a**) after implementing image preprocessing (black background, cropping, re-centering, horizontally flipping, brightness, and contrast equalization).

During image preprocessing, the input images are adjusted to fit a resolution of 1024 × 1024 by padding (adding background) or cropping the images when necessary. All images with right feet are horizontally flipped to match left-foot images. Some images require brightness and contrast modifications to ensure the bones are clearly visible. An example of an input image before and after image preprocessing is shown in Figure 3a and Figure 3b, respectively.

To evaluate the models during training, ten-fold cross-validation is used to split the dataset. Ideally, the first nine folds are used to train the model, while the holdout 10th fold is used for testing. Repeatedly, each of the folds is given an opportunity to be used as the testing set. As a result, 10 models were fitted and evaluated, and the overall performance of the model is calculated as the mean of these runs. The testing performance was evaluated by calculating the precision, recall, specificity, accuracy, and F-Score for each foot type (normal, mild flat foot, and moderate flat foot). Lastly, the overall accuracy was calculated for the model using the accuracy_score from the Skelearn library; it compares the predicted classes against the ground truth classification for each angle, and then the overall accuracy was obtained by finding the total average. For each foot type, the precision shows how good the model is at predicting a foot type, and it is calculated as the ratio of correct positive predictions (true positive TP) out of all positive predictions made (including the incorrect ones as false positive FP):

$$precision\;\left(\%\right)=\frac{TP}{TP+FP}\times 100$$

The recall (aka sensitivity) shows how many times the model was able to detect a foot type; it is calculated as the ratio of correct positive predictions out of all correct predictions made plus the ones that were supposed to be this foot type but were misclassified (false negative FN):

$$recall\;\left(\%\right)=\frac{TP}{TP+FN}\times 100$$

The specificity of a foot type measures how well the model can correctly identify the other foot types; it can be calculated as the ratio of correct negative predictions out of the sum of the correct negative predictions (true negative TN) plus the ones that are supposed to be other foot types but were classified as the current type:

$$Specificity\;\left(\%\right)=\frac{TN}{TN+FP}\times 100$$

The accuracy of a foot type is the ratio of all correct positive and negative predictions out of all predictions made.

$$Accuracy\;\left(\%\right)=\frac{TP+TN}{TP+FN+FP+TN}\times 100$$

Calculating the F-Score of a foot type can sometimes give a better assessment of a model than the accuracy rate; it helps to understand the distribution of the dataset and whether the data are balanced or not. It can also penalize the model with too many false negatives. It is calculated as:

$$F\_Score=\frac{2\left(Precision\;\times \;Recall\right)}{Precision+Recall}$$

The error rate is used in this research to investigate the impact of using a combination of three different foot angles versus a single angle. For the classifier model, the final estimations of each foot angle were accumulated and used to find the error rates as follows:

$$Error_{Rate}\left(\%\right)=\frac{FN+FP}{TP+FN+FP+TN}\times 100$$

### 3.1. Template Matching

**Figure 4:**
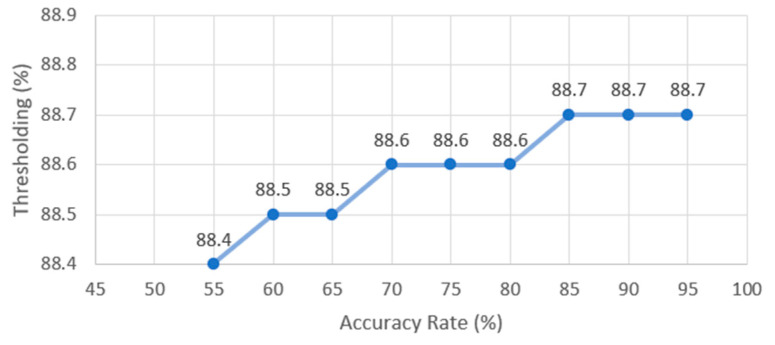
Shows the accuracy rates for Template Matching using the TM_SQDIFF_NORMED method using different thresholding values.

Several trials were conducted to select the Template Matching method that achieved the best similarity score. Among the six Template Matching methods, TM_SQDIFF_NORMED is selected with a threshold value of 90%. The thresholding value was selected after comparing different values from 50% to 95%; the comparison results are represented in Figure 4. The overall accuracy rate represents the mean similarity rate between the resulted image part and the correct image part that was manually selected (the ground truth) using the formula:

$$\frac{1}{1+d\left(GT,\;IP\right)}$$

where the distance *d* is the Euclidean distance between the ground truth image (*GT*) and the image part (*IP*):

$$d\left(GT,IP\right)=\;\sqrt{{\left(GT_{x}-IP_{x}\right)}^{2}+{\left(GT_{y}-IP_{y}\right)}^{2}}$$

After implementing Template Matching for all input images, the output is a set of potential image parts for each PoI (a total of nine sets). There is no fixed number of image parts for each PoI; it totally depends on the matching threshold. The best match has the minimum squared difference value (the highest similarity score). 

### 3.2. CNN Classifier

Since there are several image parts resulting from Template Matching, the classifier is needed to select only one image part for each PoI in every input X-ray image to form the foot angles. This selection is based on the likelihood computed by the classifier model. The feature extractor part of the model is the same for CNN and Random Forest classifiers; it consists of several convolutional layers constructed from scratch: a convolutional layer with 32 filters, a kernel size of 3 × 3, and a he_uniform kernel initializer. Followed by a batch normalization layer and a max pooling layer. Since this network was defined from scratch, it needs to start with initial weights and then iteratively update them to better values. Thus, the kernel initializer defines the way to set these initial random weights for the network. Next, the same series of layers is repeated while changing the filter size to 64. Lastly, a flattened layer was added to decrease the feature dimensions into 1-D to be input into a dense layer (a fully connected layer that is part of the CNN classifier).

The CNN classifier model consists of five DENSE_LAYERS connected to the feature extractor; the first four of them have a filter size of 128 each and the very same parameters as the feature extractor’s convolutional layers, while the last DENSE_LAYERS (the prediction layer) have a filter size of nine (number of PoIs), and the activation is softmax since it is a multiclass classification task. Several experiments were conducted for comparing the overall accuracy rate with different numbers of the DENSE_LAYERS (between two, five, and nine layers), only the last DENSE_LAYERS being the same, different epochs (between 200, 250, 300, 350, 400, and 450), and two optimization methods: RMSProp (Root Mean Squared Propagation) and ADAM (Adaptive Moment Estimation) optimizers with a categorical_crossentropy loss function, a learning rate of 0.0001, and a batch size of 32. Table 2 summarizes the results from the experiments conducted based on the type of optimizer used each time. Furthermore, for each number of DENSE_LAYERS, a variation number of epochs was used and grouped as a range from 200 to 450, and the mean accuracy rate was reported.

The results show the best mean accuracy of CNN classifiers using RMSProp has reached 68.8% and 71.3% using the RMSProp optimizer. There are some key differences between these two optimizers: RMSProp optimizer aims to reach the minimum of the loss function as fast as possible by increasing the step size (learning rate), while ADAM rapidly updates the step size and focuses on the best direction to converge towards the global minimum in fast steps. These are the best results that can be achieved using the limited dataset at hand.

In the context of deep learning, large datasets consisting of thousands of samples are required for feature extraction as well as training the models [13]. Alternatively, when the dataset at hand is smaller than enough, it is possible to load the features that have been extracted previously from huge benchmark datasets using high-performance hardware. This approach is known as transfer learning, and with it, the benefit of decreasing the training time while improving overall performance can be achieved [15]. VGG-16 [19] is an example of such pioneering CNN models that were extensively used for transfer learning because of their performance. The network was developed by the Visual Geometry Group (VGG) at the University of Oxford for image classification tasks. It was pre-trained on the ImageNet [35] dataset benchmark, which consisted of 14 million images belonging to 1000 classes. According to the source [19], the use of pre-trained models varies from one task to another; one such task is to use them as stand-alone feature extractors. The pre-trained model is loaded with some of its layers, depending on the desired types of features to be loaded. The set of layers that are closer to the input layer learn low-level features such as lines, corners, edges, etc.; the next subsequent layers learn complex abstract features that combine the lower-level features extracted from the input, such as shapes and textures; and lastly, higher-level features such as the structure of the organs will be learned by layers closer to the output in the context of a classification task. Hence, if the new task is quite different from the classification task, then only the first few layers closer to the input layer would be appropriate; otherwise, when the new task is quite similar to the classification task, then more deeper layers prior to the output layer could be used. After selecting the layer sets, the last fully connected layer and the final classification layer of the loaded pre-trained model are replaced with another two layers: a new fully connected layer that specifies the new number of classes in the new dataset, and a new classification layer that specifies the new classification labels. When training, all loaded layers are frozen and set to non-trainable, and only the new layers are trained with the new dataset (last two layers).

In the next experiment, the feature extraction part (convolutional layers) is replaced with a pre-trained model (VGG-16). All layers, from the input layer to the last MaxPooling layer of the VGG-16, are loaded; with this, no DENSE_LAYERS are included. The loaded weights are frozen, and the input shape is changed to accept the dimensions of the dataset at hand. All input images are then fed to this feature extractor to train the classifier. The CNN classifier (the same classifier used in previous experiments) uses the same settings (hyperparameters) as previous attempts. From the results in Table 3, the mean accuracy of the CNN classifier after changing the feature extractor shows improvement from 68.8% to 73.8% using RMSProp and from 71.3% to 75.0% using the ADAM optimizer. From this, using the ADAM optimizer for the CNN classifier is shown to be more effective than using RMSProp for this research. Additionally, using 200 epochs and 5 DENSE_LAYERS is enough for the classifier to achieve the best accuracy rates.

In the next experiments, augmentation is implemented to increase the dataset at hand using rotation (between −15 and 15 degrees) and scaling (up to ×1.5 scale); accordingly, the number of templates is also increased. As a result, the number of images was increased to 800: 300 normal feet, 300 mild flat feet, and 200 moderate flat feet. Using the previous settings of the feature extractor and ADAM optimizer with a fixed number of epochs (200 epochs) and 5 DENSE_LAYERS, the highest mean accuracy rate was 86.4%. 

### 3.3. Random Forest Classifier

Using the same convolutional layer-based feature extractor, the Random Forest classifier was defined with 100, 150, and 200 trees while varying the random state between 32, 42, and 48. After feature extraction, feature vectors were fed to a Random Forest classifier for training. The following Table 4 summarizes the results from the conducted experiments. For each random state, variations in the number of trees were defined as a range from 100 to 200, and the mean accuracy rate is reported. Compared to the CNN classifier, the best mean accuracy rate of the Random Forest classifier was 77.5%, which outperformed the best mean accuracy rate achieved by the CNN classifier using ADAM (71.3%). 

Table 5 shows results using the same Random Forest classifier’s variations with VGG-16 as the new feature extractor. The classifier shows improvement from 77.5% to 86.25%, which outperformed the best of CNN (75.0%). The results also show that using 42 random states is enough to achieve the best training accuracy rate. In addition, there are no major differences in the accuracy rate after using more than 100 trees.

After increasing the images using augmentation, a Random Forest classifier was used with 42 random states and 100 trees, achieving a mean accuracy rate of 94.24%, which is higher than using a CNN classifier (86.4%). The classifier was able to correctly classify 732 images (281 normal feet, 279 mild flat feet, and 172 moderate flat feet), while 68 were misclassified. In these 68 images, most of the misclassified images were moderate flat-foot cases. Among the three foot angles, the AA was the most disagreeable with the rest, while both Meary’s Angle and the CIA showed strong agreement in deciding the final estimations. There was no result where all angles reached different estimations; at least two angles agreed on the same estimation.

After training the two models, the testing set retrieved from LERA is augmented and used for comparison. Using the same augmentation configurations as in training, the testing set increased from 77 to 240 cases, including 90 normal, 75 mild flat foot, and 75 moderate flat foot. Random Forest recorded a 93.13% mean accuracy rate, outperforming the CNN model (83.9%). The following Table 6 summarizes the testing results using CNN and Random Forest classifiers for each foot type. The precision of the normal cases indicates that both models performed well in detection; however, Random Forest was able to detect more cases than CNN for the mild and moderate flat feet. Recall (sensitivity) could be a more important metric than precision; it is more desirable for a model designed for medical cases to have as few false negatives as possible. For instance, when a moderate flat foot was classified as mild, the patient’s case would worsen because they were not receiving the appropriate care planned for a moderate flat foot. The results show the Random Forest model is more sensitive to detecting all foot types than CNN. From the specificity, both classifiers can easily recognize (specify) normal cases more than flat foot cases, and both models found difficulties in recognizing mild flat foot. The accuracy of the models shows how each classifier performs generally, while F-Score considers the false negatives. From this, CNN has more false negatives than Random Forest in predicting every foot type, which means fewer cases were missed using the latter classifier.

To investigate the impact of using a combination of three different foot angles against using a single angle, each angle’s error rates were calculated during the previous experiments. Table 7 summarizes the Mean Error Rate (MER) for each foot angle after reaching the final estimation using CNN and Random Forest classifiers. From the results, the Arch Angle shows the highest error rates among the foot angles, and most of the errors occurred when detecting mild flat foot cases. One reason might be that the selected angle interval for the Arch Angle from the selected sources was not accurate. To prove this reason, another angle interval from a different source could be used. Another possible reason is that the PoIs that define the Arch Angle might not be accurately allocated.

## 4. Discussion

Flat feet are a common deformity that affects millions of people globally. It is either congenital or acquired from accidental injuries; it is also common to witness flat feet with diabetes and other foot disorders [36,37]. Mainly, a flat foot alters the natural reflexes of the sole, which affect posture and gait [37,38], both of which play an important role in balance and proprioception. Affecting the natural posture means limiting the ability to maintain an upright stance while welling, while affecting the natural gait refers to performing unstable movements [39,40]. If the flat foot persists without attending treatments at the time, several consequences can occur, including stressful pain, muscle weakness, and advanced functional disabilities, which negatively affect an individual’s life [41]. Therefore, detecting the deformity before developing the risks as well as precisely determining its severity levels to attend to the proper treatment plan may prevent such an impact on the individual’s quality of life.

The aim of this research was to develop an automated model that detects flat foot cases and their severity levels from lateral foot X-ray images by measuring three different foot angles: the Meary’s Angle and the Calcaneal Inclination Angle. These angles basically depend on a set of Points of Interest (PoIs) on the image. Template Matching is used to find a set of potential PoIs for each angle, and then a classifier is used to select a single point that has the highest predicted likelihood. These points are connected, and the formed angle is measured and compared to the angle intervals to estimate the foot case. Two different classifiers were constructed: a Convolutional Neural Network-based model (CNN) and a Random Forest-based model. The major factor affecting the overall performance was the size of the dataset. Since the collected images for this research are considerably small, two solutions were suggested: using transfer learning as a feature extractor and image augmentation. As a result, the overall accuracy rate increased from 86.25% to 93.13% using the Random Forest model and from 75.0% to 83.9% using CNN with the same VGG-16 feature extractor in both models.

After investigating the effect of using a combination of three different foot angles instead of a single angle, this approach helps when two of the three angles show agreement in the final estimation while the remaining angle disagrees. In such cases, the results of these two angles are considered. Among the used foot angles, the AA recorded the most disagreement counts with the other angles in reaching the final estimation. Among the selected foot angles for this research, the AA showed disagreement with the other angles in most of the cases. Most of the cases in which the angles disagreed on its final estimation were its severity levels too close to their next level, like when a normal foot was almost flattened but not too flat to fall within the flat foot category. In such cases, AA made the most of the incorrect estimations, and if the model were to rely solely on AA, then it would affect its accuracy rate.

It is possible to replace Template Matching with different matching-based methods using the same template set in this research, such as Scale Invariant Feature Transform (SIFT) or Speeded Up Robust Features (SURF) descriptors, part of the openCV function from the Features2D Framework. Alternatively, it is also possible to use object detector models to directly find the bones of interest in the input image and then apply some mathematical operations to find the exact locations of the points of interest. 

The proposed work is limited by the lateral view of the X-ray foot images and all flat foot severity levels, excluding rigid cases (more advanced cases of flat foot). In addition, all the collected X-ray images and the measured foot angles were for adults (18 and above), and children (juvenile flat feet) should have different measurements and conditions.

## 5. Conclusions

After conducting several extensive experiments on the collected dataset at hand, the proposed method in this work provided promising results, and several findings could lead to important advances in subsequent research, including: (1) Using transfer learning techniques as a feature extractor only, in addition to image augmentation, would greatly increase the overall accuracy rate. (2) Relying on three different foot angles shows more accurate estimations than measuring a single foot angle. It is hopefully possible to extend the proposed method to include other foot angles for comparison as well as implement it on rigid flat feet in the future.

## Figures and Tables

**Table 1:** Foot angle interval measurements in degrees.

Angle	Normal Range	Mild Flat Foot	Moderate Flat Foot
CIA	20 ≤ a ≤ 30	10 ≤ a < 20	10 > a
AA	150 ≤ a ≤ 165	170 < a < 180	180 ≤ a
MA	0 < a < 4	4 ≤ a ≤ 14	15 ≥ a

Notes: foot angles: CIA: Calcaneal Inclination, AA: Arch, MA: Meary’s Angle, a: measured foot angle.

**Table 2:** Results summary for several experiments using a CNN classifier for hyperparameter tuning.

Optimizer Method	# of Dense_Layers	# of Epochs	Mean Accuracy Rate (%)
RMSProp	2	200–450	61.3
	5	200–450	68.8
	9	200–450	68.8
ADAM	2	200–450	63.8
	5	200–450	71.3
	9	200–450	71.3

**Table 3:** Results summary for CNN classifier using VGG-16 as feature extractor.

Optimizer Method	# of Dense_Layers	# of Epochs	Mean Accuracy Rate (%)
RMSProp	2	200–450	70.0
	5	200–450	73.8
	9	200–450	73.8
ADAM	2	200–450	71.3
	5	200–450	75.0
	9	200–450	75.0

**Table 4:** Results summary using a Random Forest classifier while varying random states and the number of trees.

Random State	# of Trees	Mean Accuracy Rate (%)
32	100–200	72.5
42	100–200	77.5
48	100–200	77.5

**Table 5:** Results summary using Random Forest classifier with VGG-16 as feature extractor.

Random State	# of Trees	Mean Accuracy Rate (%)
32	100–200	82.5
42	100–200	86.25
48	100–200	86.25

**Table 6:** Summary of test results using CNN and Random Forest classifiers.

Foot Type	Precision	Recall	Specificity	Accuracy	F-Score
CNN Classifier					
Normal	96.55	93.33	98.00	96.25	94.92
Mild	81.81	90.00	88.00	88.75	85.71
Moderate	88.89	80.00	96.67	92.50	84.21
Random Forest Classifier					
Normal	97.62	95.67	98.60	97.50	96.63
Mild	88.40	94.00	92.60	93.13	91.12
Moderate	94.12	88.00	98.167	95.63	90.96

**Table 7:** Foot Angle Mean Error Rates (MER) using CNN and Random Forest.

Foot Type	Arch Angle	Meary’s Angle	Calcaneal Inclination Angle
CNN Classifier			
Normal	15.28	14.69	14.00
Mild	20.45	17.66	17.66
Moderate	16.87	15.20	13.34
Random Forest			
Normal	10.12	7.08	6.18
Mild	11.13	7.37	6.69
Moderate	10.61	7.32	7.83

## Data Availability

The data used in conducted experiments is unavailable for public due to privacy.
